# Correction: Healthcare providers as patients: COVID-19 experience

**DOI:** 10.1371/journal.pone.0305372

**Published:** 2024-06-06

**Authors:** Abbas Al Mutair, Alexander Woodman, Amal I. Al Hassawi, Zainab Ambani, Mohammed I. Al Bazroun, Fatimah S. Alahmed, Mary A. Defensor, Chandni Saha, Faiza Aljarameez

The affiliation for the fourth author is incorrect. Zainab Ambani is not affiliated with #7 but with #8: King Saud bin Abdulaziz for Health Sciences, King Abdullah International Medical Research Center, Ministry of the National Guard Health Affairs, Al-Hasa, Saudi Arabia.
